# HLA-dependent tumour development: a role for tumour associate macrophages?

**DOI:** 10.1186/1479-5876-11-247

**Published:** 2013-10-06

**Authors:** Maddalena Marchesi, Emilia Andersson, Lisa Villabona, Barbara Seliger, Andreas Lundqvist, Rolf Kiessling, Giuseppe V Masucci

**Affiliations:** 1 Department of Oncology-Pathology, Karolinska Institutet, Karolinska University Hospital, SE-171 76 Stockholm, Sweden; 2 Institute of Medical Immunology, Martin Luther University Halle-Wittenberg, Halle/Saale, Germany; 3 Roche Pharma, Basel, Switzerland

## Abstract

HLA abnormalities on tumour cells for immune escape have been widely described. In addition, cellular components of the tumour microenvironment, in particular myeloid derived suppressor cells (MDSC) and alternatively activated M2 tumour-associated macrophages (TAMs), are involved in tumour promotion, progression, angiogenesis and suppression of anti-tumour immunity. However, the role of HLA in these activities is poorly understood. This review details MHC class I characteristics and describes MHC class I receptors functions. This analysis established the basis for a reflection about the crosstalk among the tumour cells, the TAMs and the cells mediating an immune response.

The tumour cells and TAMs exploit MHC class I molecules to modulate the surrounding immune cells. HLA A, B, C and G molecules down-regulate the macrophage myeloid activation through the interaction with the inhibitory LILRB receptors. HLA A, B, C are able to engage inhibitory KIR receptors negatively regulating the Natural Killer and cytotoxic T lymphocytes function while HLA-G induces the secretion of pro-angiogenic cytokines and chemokine thanks to an activator KIR receptor expressed by a minority of peripheral NK cells. The open conformer of classical MHC-I is able to interact with LILRA receptors described as being associated to the Th2-type cytokine response, triggering a condition for the M2 like TAM polarization. In addition, HLA-E antigens on the surface of the TAMs bind the inhibitory receptor CD94/NKG2A expressed by a subset of NK cells and activated cytotoxic T lymphocytes protecting from the cytolysis.

Furthermore MHC class II expression by antigen presenting cells is finely regulated by factors provided with immunological capacities. Tumour-associated macrophages show an epigenetically controlled down-regulation of the MHC class II expression induced by the decoy receptor DcR3, a member of the TNFR, which further enhances the M2-like polarization. BAT3, a positive regulator of MHC class II expression in normal macrophages, seems to be secreted by TAMs, consequently lacking its intracellular function, it looks like acting as an immunosuppressive factor.

In conclusion HLA could cover a considerable role in tumour-development orchestrated by tumour-associated macrophages.

## Introduction

Macrophages are tissue phagocytic cells that display the main activity of clearance independently of immune cells. However, they can be activated in response to either innate or adaptive immune signals and they are able to shape their phenotype to fit the microenvironment. In an attempt to simplify their complexity, activated macrophages can be categorized into two distinct subtypes: classically activated (M1) and alternatively activated (M2) [1,2]. M1 macrophages are activated in the presence of bacterial moieties (LPS) or Th1 cytokines (IFN-gamma). In mice, M1-associated markers include interleukin-12 (IL-12), inducible nitric oxide synthase 2 (iNOS2) and a high level of MHC class II molecules. Human macrophages do not show induction of iNOS2 under the same conditions, despite having a similar phenotype. They have an efficient antigen presentation capacity and, through interaction with Th1 cells and NK cells, they are able to kill pathogens and tumour cells. M1 macrophages antagonize tumourigenesis due to direct tumour-killing mechanisms and amplification of Th1 responses, providing a positive feedback loop in the anti-tumour response [1]. In contrast, M2 macrophages are activated in the presence of Th2 cells cytokines and basophil-secreted cytokines (IL4, IL13, IL33) or induced by immune complexes and TLR agonists as well as by IL-10 and glucocorticoids [3-5]. The expression of resistin-like alpha (FIZZ1), arginase-1, macrophage mannose receptor 1 and chitinase 3-like 3 identify mice M2 macrophages, while human M2 macrophages do not show induction of resistin-like-alpha, arginase 1 and chitinase 3-like 3 but they up regulate indoleamine 2,3-dioxygenase (IDO). Moreover, M2 macrophages produce several growth factors (IL-10, EGF, FGF, VEGF and TGF-beta) and they are also involved in parasite clearance and allergy. M2 macrophages may enhance tumour progression by releasing growth factors and products of the arginase-1/IDO pathway and by promoting tumour angiogenesis [6].

Solid tumours are infiltrated by a heterogeneous immune cell population, including different subsets of lymphoid and myeloid cells [7]. The infiltration of tumour-associated macrophages (TAMs) can represent an index of poor prognosis in different types of cancer [8].

Mantovani and collaborators, as a result of a literature review, suggest a switch of TAMs from M1-like to M2-like phenotype as an adaptation to microenvironment changes during tumour progression. In early carcinogenesis T cells drive M1-activated macrophages to eliminate tumour cells except in advanced stages of tumour progression where microenviromental signals result in M2 polarized TAMs that orchestrate smouldering, non-resolving tumour promoting inflammation [9,10].

This fine property of TAMs to modulate their features in relation to different microenviromental conditions is clearly shown by Movahedi K. and co-authors [11]. Using a mammary adenocarcinoma model, they provide a dynamic picture in which a subset of precursor monocytes infiltrating the tumour gives rise to distinct TAM subsets. These subsets are different at the molecular and functional levels and were present in different intra-tumour microenvironments. TAMs with a high expression of MHC class II and M1 markers are confined in normoxic tumour areas, whereas M2-like TAMs with a low expression of MHC class II and significantly higher proangiogenic activity are found in hypoxic tumour areas. Moreover, these M2-like TAM subsets possess a poor antigen presenting capacity and could suppress T-cell activation by using different suppressive mechanisms such as: the secretion of protease like Arg-1 [12], the releasing of immunosuppressive cytokines like TGFbeta [8] and IL-10 [13] and of chemokine-like CCL18 [14] the membrane expression of B7-H1 [15] and BTN3A [16]. Importantly, the relative percentages of these distinct myeloid subpopulations dramatically changed as tumours progressed: the TAM subset expressing low MHC class II became gradually the more represented population of the myeloid tumour infiltrate [14].

The alterations of MHC class I molecules and cellular components involved in the antigen processing and presentation machinery have been widely described as one of the main mechanism adopt by various type of tumour cells to escape from the immune system. The frequency of the HLA class I abnormalities is highly variable in many solid and hematopoietic tumours.

These deviations include the complete absence or down-regulation of HLA class I expression or of HLA class I allo-specificities in frequent association to an impairment of a single or various members of the antigen processing and presenting machinery. In most cases, it seems that the HLA class I abnormalities result from a deregulation rather than a structural defect suggesting an intervention at the epigenetic, transcriptional and or posttranscriptional level [17]. This observation increase even more the importance of studying the crosstalk between the tumour cells and the microenvironment.

This review tries to provide a snapshot of the complex and dynamic scene of cancer HLA is the focus, but the subject matter has been broadened to include the tumour cells, the microenvironment and their interaction.

### Classical and non-classical MHC class I

#### HLA-A, -B, -C

HLA-A and HLA-B antigens are constitutively expressed on macrophages and are not altered during macrophage differentiation [18], whereas HLA-C antigens are constitutively expressed at much lower levels. The reason for this is based on the necessity of maintaining the NK cell activation at a threshold that assures immunological surveillance. The regulation of HLA-C expression occurs at different steps of the biosynthesis pathway [19,20]. The cytoplasmic tail of HLA-C contains internalization and lysosomal targeting signals that could be inhibited upon macrophage differentiation [18]. The consequences are that undifferentiated primary monocytes, similarly to other cell types, express low levels of HLA-C, while differentiated macrophages show an up-regulation of HLA-C expression.

The MHC class I complex can spontaneously dissociate and be released as Beta2-microglobulin free form by activated T lymphocytes [21], or can escape from the ER as free heavy chain Beta2-microglobulin and appears as a beta2-microglobulin-free form on the tumour cell surface [22].

Consequently, MHC class I molecules are present on the cell surface in two forms: one is the Beta2-microglobulin-associated form and the other is a free heavy chain (HC) with an open conformation. The equilibrium between these conformations on the cell surface can be modified by the activation state or by transformation [21]. The free HC of HLA-C molecules are commonly observed on normal cells like activated lymphocytes [23] and are stably expressed by melanoma cell lines [22].

Thus, HLA-C is characterized by a low affinity for Beta2-microglobulin and, therefore, it might be significantly expressed on the cell surface in the free heavy-chain form of activated macrophages [18]. The hypothetical role of HLA-C, both the B2-microglobulin associated form and the free HC form has still to be explored.

#### HLA-G

HLA-G consists of seven isoforms (four membrane-bound: from HLA-G1 to HLA-G4 and three soluble: from HLA-G5 to G7) that are generated by alternate splicing. In addition, soluble HLA-G could be shed by the proteolytic cleavage of membrane bound isoforms. The isoforms mainly described, the cell-surface HLA-G1 and the soluble form HLA-G5, are detected as Beta2-microglobulin-free heavy chains and as disulphide-bound homodimers and homotrimers [24,25]. Zilberman et al. identify both surface and soluble HLA-G5 dimers in tumour cells and malignant ascites [26].

Multimeric MHC class I molecules in most cases are able to induce a more potent immune response in comparison with monomers [27]. In fact, dimers of HLA-G1 and HLA-G oligomers bind with increased affinity the inhibitory receptors of the leukocyte immunoglobulin-like family (ILT2 and ILT4) than monomers, resulting in an enhanced inhibitory function of myeloid cells *in vitro*[25,28,29] and *in vivo*[30,31].

The different isoforms of HLA-G have been shown to be expressed both by the tumour cell and by tumour associated macrophages/monocytes in lung cancer, melanoma, breast cancer and neuroblastoma [32-35] and by glioblastoma infiltrating microglia [36]. In addition, soluble HLA-G can be secreted in the peripheral blood of cancer patients [37-44]. An interesting study shows that soluble factors released by neuroblastoma cells could reach the blood stream and induce a programme of “frustrated activation” with features of macrophage-like activated cells in systemic monocytes. Thus, the release of higher amounts of immune-suppressive soluble HLA-G and less IL12 indicates a shift towards a more anergic phenotype [45].

#### HLA-E

HLA-E surface expression requires the expression of certain MHC class I molecules including HLA-A, HLA-B, HLA-C, and HLA-G (not HLA-F), which possess a nonameric peptide in their leader sequence capable of binding to HLA-E. This is usually co-expressed in HLA-G positive tissue [46,47]. HLA-E cell surface expression is limited to extra villous trophoblast cells, endothelial cells of all types of vessels, B- and T lymphocytes, NK cells, monocytes and macrophages [48].

Moreover, HLA-E is expressed by tumour cells of the following cancers: colorectal, laryngeal, ovarian, breast, melanoma, lymphoma and glioma [49-56]. The observed association of antigen processing defects, down-regulation of Beta2-microglobulin or other MHC I heavy chain genes and induction of HLA-E in malignant tissues suggests that HLA-E expression becomes independent by the action of the nonameric peptides expression [57]. The nonameric peptides are replaced by peptides derived from self-proteins [58] that confer invisibility by NK lysis [59].

HLA-E could be released in soluble forms by melanoma cells [54,60]. Kren L. et al. detected HLA-E expression on tumour infiltrating microglia/macrophages in most cases of glioblastomas analysed, while HLA-E expression is lacking in normal microglia [36]. Since most tumour infiltrating microglia/macrophages are found at the border of the ischaemic area, hypoxic stimuli enhance the expression of the HLA-E and HLA-G-immune modulatory molecule [61] as far as it induces M2 polarization [11].

#### HLA-F

HLA-F is the least characterized member of the non-classical HLA class I molecules. It is intracellularly retained by almost all resting leukocytes, except B lymphoblastoid- and monocytic cell lines which express it on the cell membrane [62] but it may reach the cell surface under activation. HLA-F is not up-regulated in CD4+, CD25+ FoxP3+ regulatory T cells when they are activated, whereas, under identical conditions, CD4+, CD25- T cells show strong surface HLA-F expression. HLA-F could provide a signal that indicates an activated immune response to regulatory T cells and it could trigger the secretion of immunosuppressive cytokine leading to tolerance [63]. HLA-F is suggested as an unfavourable prognostic factor in patients affected by NSLC [64] and by oesophageal squamous cell carcinoma [65]. In addition, anti-HLA-F IgG is present in the sera derived from various types of cancer patients, but not in the sera derived from healthy donors [66].

HLA-F expression is entirely dependent on its cytoplasmic tail to exit from the endoplasmic reticulum. Although it is not known if this pathway requires peptide binding, it is conceivable that export of HLA-F from ER may be a peptide independent pathway [67] even though HLA-F can bind to TAP molecules [68]. Consequently, it is expressed both as a Beta2-microglobulin-free HC form independently from TAP and as a heterodimer form with Beta2-microglobulin [62]. HLA-F surface expression is strictly dependent on the expression of MHC class I HC as far as it is downregulated upon perturbation of the same molecule. Moreover, MHC class I is able to interact with HLA-F only when it is in the form of an open conformer free of peptides. All these data suggest that the physical cis-interaction plays a role in the function of MHC class I molecule in activated immune cells [69].

The surface plasmon resonance studies show that tetrameric complexes of HLA-F are able to interact with affinity to the inhibitory receptors of the leukocyte immunoglobulin-like family ILT2 and ILT4 [70]. The emerging questions are, what are the roles of HLA-F on TAMs and of its interaction with MHCclass I molecules and with the receptors ILT2 and ILT4? The structure of classical and non classical MHC class I molecules is simplified in the Table 1.

**Table 1:** Summary of structure of MHC class I molecules and their receptors

**MHC class I**	**CELL TYPE**	**Beta2 microglobulin associated**		**Open conformer form/RECEPTORS**	
		**form/RECEPTORS**			
HLA A, B	Nearly all cells	+++	ILT2 ILT4 KIR3DL1/2	+/−	
	Tumor cells	+++/++/+	ILT2 ILT4 KIR3DL1/2	+++/++/+	ILT4 KIR3DL1/2
	TAMs	?		?	
HLA C	Nearly all cells	+	ILT2 ILT4 KIR2DL1/2/3	+/−	
	Activated immune cells	+/−		+++	ILT4 LILRA KIR2DL1/2/3
	Tumor cells (melanoma)	+/−		+++	ILT4 LILRA KIR2DL1/2/3
	TAMs	?		?	
HLA G	Fetal derived placental cells	+++	ILT2 ILT4 KIR2DL4	?	
	Tumor cells (colorectal/laryngeal, ovarian/breast carcinoma, lymphoma, glioma, melanoma)	+		+++ (monomer/ dimer/trimer)	ILT4 KIR2DL4
	TAMs	+		+++ (monomer/ dimer/trimer)	ILT4 KIR2DL4
HLA E	Extravillous trophoblast cells	+++ (MHC-I derived peptide)	NKG2A	?	
	Endothelial and immune cells	+++ (MHC-I derived peptide)	NKG2A	?	
	Tumor cells	+++ (self peptide)	NKG2A	?	
	TAMs	+++ (self peptide)	NKG2A	?	
HLA F	Resting immune cells	-		-	
	Activated immune cells	+++	ILT2 ILT4	+++	ILT2 ILT4
	Tumor cells (NSLC and esophageal carcinoma)	+++	ILT2 ILT4	+++	ILT2 ILT4
	TAMs	?		?	

### MHC class I receptors

#### Leukocyte immunoglobulin-like receptors

Leukocyte immunoglobulin-like receptors (LILRs) are HLA receptors widely expressed by myeloid cells and their role as modulators of macrophage activation has been well described [71,72]. Their importance in tumour immune escape can be foreseen by the observation that LILRs are exploited by viruses to evade from the immune system. A clear example is shown by a study conducted on HIV variant showing that a mutated immunodominant virus epitope linked to HLA-B2705 creates a better ligand for ILT4 on myelomonocytic cells and this interaction leads the antigen presenting cells (APCs) to a tolerogenic profile [73]. In a similar manner, CMV encodes epitopes which, linked to MHC class I molecules, is able to increase affinity to ILT2 compared with regular MHC class I molecules reducing antiviral immune surveillance [74].

The LILR family includes inhibitory (LILRB) and activating (LILRA) receptors. The best-characterized LILR members remain the inhibitory receptors LILRB1 (ILT2) and LILRB2 (ILT4). ILT2 is widely expressed on myeloid and lymphoid cells, while ILT4 is almost exclusively expressed on monocytes, macrophages and dendritic cells. ILT2 and ILT4 bind *in trans* with a broad specificity [75] both classical and non-classical HLA class I molecules. These recognize the non-classical HLA-G with a higher affinity [76,77]. Moreover, ILT2 cannot recognize the Beta2-free form of MHC class I [77]. In contrast, ILT4 can bind the Beta2-microglobulin-free classical MHC class I molecules, like HLA-B27 [78], HLA-C [79] as well as the non-classical Beta2-microglobulin-free HLA-G molecules [77]. This *in trans* interaction has a different affinity between the soluble forms of ILT2 and ILT4 and several MHC class I molecules [80]. Jones et al. studied LILRs binding to the products of >90 different HLA class I alleles [79]. ILT2 and ILT4 were linked to different HLA alleles with different strength in relation to amino acid motifs within the alpha3 region (polimorphisms) and HLA I class conformation. The ligation of individual HLA class I alleles to ILT2 and ILT4 may alter the signalling through the inhibitory receptor and consequently the level of activation of APCs. Actually, HLA allele B*3503 binds ILT4 significantly stronger than other HLA class I molecules do, independently of the presented epitopes [81]. Consequently, HIV-1-infected, HLA B*3503 carriers have APCs with poor functional properties and an accelerated HIV-1 disease.

Among activating receptors, LILRA1 marks monocytes, LILRA2 is expressed on monocytes, macrophages, neutrophils, eosinophils and basophils [82]. LILRA3 is a homologue of LILRA1 but lacks the transmembrane domain and is therefore a secreted molecule, mainly produced by macrophages [83]. These recognize MHC class I molecules [75]. On the other hand, LILRA1 and LILRA3 display a preference for specific alleles Beta2-microglobulin-free forms [79], most of which belong to the HLA-C locus [84].

While the affinity of ILT4 for free heavy chains of different MHC class I antigens results in a slightly reduced than completed MHC-I form, LILRA1 and LILRA3 only recognize open conformer, classical and non-classical HLA class I antigens, especially HLA-C alleles [79,85]. LILRA1 binding to HLA-B27 has also been reported [78]. Another strong ligand for LILRA1 and LILRA3 is the free form of HLA-B*0801 [79], a component of the MHC 8.1 ancestral haplotype associated with autoimmune conditions mainly due to an exaggerated Th2-type cytokine response [84]. This haplotype is also predictive of a shorter progression free and overall survival in non-Hodgkin lymphoma [86] and in malignant melanoma [87].

We can suppose that macrophages dipped in tumour microenvironment and pushed by Th2-type cytokines, due to the interaction between LILRA and the complex ancestral haplotype-cancer antigen-differentiate in M2-like macrophages. Hence, this could explain how MHC 8.1 haplotype could work as a prognostic factor in patients affected by NHL and melanoma.

LILRA2 does not bind to MHC and the reason is linked to its extracellular domain, which reveals structural shifts of the corresponding MHC-binding amino acid residues in comparison with ILT2 or ILT4 [88]. This explains the lack of binding to MHC molecules. Moreover, it seems that LILRA2 forms a domain-swapped dimer. The structure described supports the dimer conformation in solution observed earlier, and implies a stress-induced regulation by dimerization. Thus, its stimulation, similar to LILRA1, induces the shift from a Th1 production (IL12) towards Th2 profile (IL-10). This is supported by an activating mutation located in the linker region of the receptor, responsible for a risk for some autoimmune diseases such as systemic lupus erythematosus and microscopic polyangiitis [82]. LILRA2 might be an immune modulator either by inhibiting DC differentiation or by stimulating an alternative macrophage differentiation pathway leading to a reduced antigen presenting capacity [89]. A role of LILRA receptors in TAMs could be postulated, but it has not yet been shown.

Moreover, the Beta2-microglobulin-free MHC I class molecules are able to *cis*-associate with LILR members modulating the receptor-mediated internalization and the signalling. The function of this interaction seems to be related to the ability of working as regulators of in *trans* ligand-receptor interactions amplifying or dampening the efficiency of the signalling [90]. The *cis*-interaction between MHC I class and ILT2 is well established in mast cells dampening cell activation [91]. No information is available on the role in TAMs.

#### Killer cell immunoglobulin-like receptor (KIR)

The Killer cell immunoglobulin-like receptor (KIR) family regulates the activation state of NK cells upon recognition of MHC class I ligands on the surface of target cells [92-94]. The inhibitor-receptors are expressed on the cell surface including KIR2DL1/2/3 and KIR3DL1/2. The first group recognizes HLA-C and the second group HLA-A and HLA-B. The activator receptor is KIR2DL4, which resides in the endosome. The only known ligand is HLA-G. Both soluble HLA-G5 and HLA-G, shed from the cell surface of transfected cells, are accumulated into endosomes that contain KIR2DL4 [95]. It is likely that the transient passage of KIR2DL4 at the cell surface, either by newly synthesized KIR2DL4 or by the recycling of endosomal KIR2DL4, is sufficient to capture soluble HLA-G and transport it to endosomes [96]. Analysis of the transcriptional response to activation via KIR2DL4 revealed the up-regulation of a restricted set of chemokines and cytokines, including IFN-gamma, TNFalfa, IL1Beta, IL6 and IL8 that can promote vascular remodelling either directly or indirectly by acting on other cell types in the local microenvironment [95,97,98].

The complex nonameric peptide-HLA-E links the heterodimer CD94/NKG2 receptor expressed by approximately 50% of NK and activated cytotoxic T lymphocytes [99]. The NKG2 family includes activating members like NKG2C and NKG2E and an inhibitory member NKG2A [100]. The complex nonameric peptide HLA-E binds the inhibitory receptor CD94/NKG2A with higher affinity than the other receptors. This determines the protection from HLA-E positive cells from NK and CTL dependent lysis [101,102].

A comprehensive summary of MHC class I antigens, their receptors and function is shown in Tables 1, 2 and 3.

**Table 2:** Summary of MHC class I molecules expressed/released on/by tumor cells, the related ligands on myeloid cells and the function of their interaction

**MHC (tumor cells)**	**RECEPTOR (myeloid cells)**	**FUNCTION**	**References**
Membrane **HLA-G1** and soluble **HLA-G5**	**ILT2** and **ILT4** on macrophages/TAMs	- Up-regulation of LILRB receptor on macrophages/TAMs	- LeMaoult J., 2005 [130]
		- Turning-down macrophage activation level	- Jones D.C., 2011 [79]
Membrane **HLA-A-B-C**	**ILT2** and **ILT4** on macrophages/TAMs	- Turning-down macrophage activation level	- Jones D.C., 2011 [79]
	**LILRA1** and **LILRA3** on macrophages/TAMs	- Stimulation of the alternative macrophage differentation	- Lee D.J., 2007 [89]
Soluble **HLA-G5**	**ILT2** and **ILT4** on macrophages/TAMs	- Secretion of TGFbeta1 resulting in blood monocytes recruitment and suppression of their cytotoxic function	- McIntire R.H., 2004 [122]
			- Ashcroft G.S., 1999 [123]
	**ILT2** on MDSC	-Expansion of MDSC with enhanced suppressive activity	- Zhang W., 2008 [124]
		-Secretion of Th2 cytokines able to skew the alternative macrophage differentation	- Agaugue S., 2011 [125]
	**ILT4** on monocytes	- Inhibition of maturation of monocyte-derived APC	- Ristich V., 2005 [129]
		- Development of tolerogenic APC	

MHC class I molecules can be used by tumor cells: to modify the function of macrophages promoting their differentation into TAMs, to interfere with monocytes maturation and to expand MDSCs which, secreting Th2 cytokines, contribute to M2 polarization of macrophages.

**Table 3:** Summary of MHC class I molecules expressed/secreted on/by macrophages/tumor associated macrophages, their related ligands on immune cells and the function of their interaction

**MHC (macrophage/**	**RECEPTOR (immune**	**FUNCTION**	**References**
**TAMs)**	**cells)**		
Membrane **HLA-A-B-C**	**ILT2** on NK cells	- Negative regulation of INFgamma secretion	- Morel E. 2008 [133]
	**ILT2** on T cells	- Negative regulation of T cell function	- Dietrich J. 2001 [136]
Membrane **HLA-A-B**	**KIR3DL1/2** on NK cells and effector T cells	- Inhibition of cytolitic function	- Anfossi N. 2004 [93]
			- Long E.O. 2008 [94]
Membrane **HLA-C**	**KIR2DL1/2/3** on NK cells and effector T cell	- Inhibition of cytolitic function	- Colonna M. 1993 [92]
			- Falk C.S. 1995 [132]
Membrane **HLA-G1**		- HLA-G1 is acquired through trogocytosis by CD4+ and CD8+ T cells resulting their temporarily reversion to regulatory T cells	- LeMaoult J. 2007 [143]
Soluble **HLA-G5**	**ILT2** and **ILT4** on myeloid APC	- secretion of the cytokine TGF-beta1 that contributes to T cells peripheral tolerance	- McIntire R.H. 2004 [122]
		- Turning down myeloid APC activation level	- Jones D.C. 2011 [79]
	**KIR2DL4** on CD56bright NK cells	- Secretion of pro-angiogenic cytokines and chemokines	- Rajagopalan S. 1999 [147]
			- Rajagopalan S. 2012 [96]
	**CD8** on activated CD8+ T cells and CD8+ NK cells	- Fas/Fas ligand mediated cell death	- Contini P. 2003 [145]
			- Puppo F. 2002 [146]
			- LeMaoult J. 2004 [144]
Membrane **HLA-E**	**CD94/NKG2A** on NK cells and cytotoxic T cells	- Production of immune-suppressive cytokines (IL-10 and TGF-beta1) by NK cells	- Jinushi M. 2004 [148]
		- Inhibition of cytolitic function	- Braud V.M. 1998 [46]
			- Borrego F. 1998 [102]

MHC class I molecules can be used by TAMs to promote tumor angiogenesis and to dampen immune response against tumor cells both directly inhibiting NK and T cells function and indirectly inducing the secretion of immunosuppressive cytokines.

### MHC class II molecules

MHC class II gene expression is exclusively governed by the master regulatory factor CIITA (class II transactivator) [103]. The MHC class II expression in TAMs is repressed by the soluble member of the TNF receptor super family, decoy receptor 3 (DcR3), through the histone deacetylation of the CIITA promoter [104]. Chang et al. propose a more complex role of DcR3 as a modulator of macrophage differentiation and as a promoter of alternatively activated macrophage polarization up-regulating genes characteristically expressed in M2 macrophages, while down-regulated genes identify M1 macrophages. Coherently in DcR3 transgenic mice, the Th1 cell-response is attenuated and deviates towards the Th2 phenotype [105] and Th2 derived cytokines skew M2-like macrophage polarization.

DcR3 is a novel immune-suppressive factor up-regulated and secreted by tumour cells like lymphoma [106], glioma [107], lung and colon cancer [108], gastrointestinal tract cancer [109], and kidney cancer [110]. It is able to down-regulate the activation of DC [111] and macrophages [112], and to induce dendritic cell apoptosis [113].

DcR3 is able to promote tumour development *in vivo* through the regulation of TAM differentiation [114]. In fact, when DcR3 is regulated by the CD68 promoter in transgenic mice, the tumour grows faster and metastasis is more common than in wild type mice. Moreover, the tumour bulk has a more elevated infiltration of DcR3-overexpressing TAMs (characterized by up-regulation of arginase activity and down-regulation of MHC class II expression). Interestingly, treatment with a histone deacetylase inhibitor can restore MHC class II expression in TAMs *in vitro*[112] and inhibit tumour growth in the transgenic model [114]. This may be of interest for future cancer immunotherapy strategies.

BAT3 is the product of a group of genes adjacent to the HLA-B locus, designated as B-associated transcripts (BATs). Kamper et al. [115] described that BAT3 regulates HLA class II expression and investigated the mode of BAT3 action on HLA class II genes. IFN-gamma, (key cytokine inducting Th1 response and M1 classical macrophage activation) modulates the expression of CIITA and strongly elevates BAT3 transcription in various tumour cell lines and in primary macrophages. Following IFN-gamma treatment, BAT3 chaperones CIITA and the complex translocates into the nuclear, where CIITA binds to the enhanceosome of HLA class II promoters enhancing HLA II gene transcription. Thus, BAT3 is critical to maintain HLA class II expression during the cell-mediated immune response. Coherently, *in vitro* exposure to INF-gamma could “re-educate” TAMs towards M1-polarized immune-stimulatory macrophages [116]. BAT3 has different intracellular roles as a chaperone: it monitors protein quality and refolding [117] and in complex with E1A-binding protein p300, BAT3 is required for the p53 acetylation in response to DNA damage resulting in DNA repair or in cell apoptosis [118]. BAT3 could be released as a BAT3 surface-positive exosome by accessory cells or tumour line cells in response to stress signals and engage NKp30, a receptor selectively expressed on NK [119,120]. The result is the NK activation and killing of accessory or tumour cells and tumour rejection in a multiple myeloma xenograft model. It is likely that other immune-suppressive factors, such as the soluble BAT3 form (purified supernatant and derived from tumour cell culture in response to non-lethal heat shock) act in a suppressive manner inhibiting NK cell-dependent cytokine release. So far, the existence of a different isoform of BAT3 that could be released in an exosomal or soluble form with a NK activatory or inhibitory function cannot be ruled out. BAT3 could be considered a sensor for DNA instability or damage through its links to p53 function, able to alert the immune system via an activating receptor on NK cells. At the same time, BAT3 is a cross talk mediator between NK and accessory cells.

In consideration of the important intracellular and immunological functions, BAT3 could have a crucial role in tumour promotion. This is corroborated by a linkage disequilibrium study suggesting BAT3 as a strong candidate for lung cancer susceptibility [121].

### Final reflections

As macrophages come into close association with tumour cells, they are exposed to high concentrations of soluble HLA-G. The binding of HLA-G5 to inhibitory receptors ILT2 and ILT4 stimulates macrophage production of TGF-beta1 [122] resulting in a further recruitment of monocytes from blood circulation and suppression of their cytotoxic function [123].

In ILT2 transgenic mice, HLA-G engagement of ILT2 receptors results in expansion of the population of myeloid derived suppressor cells (MDSC) with an enhanced suppressive activity. Moreover, ILT2 engagement results in increased production of IL-4 and IL-13, which are directly involved in the up-regulation of Arginase 1, a well-known marker of M2 macrophage polarization [124]. ILT2 ligation induces Th2 cytokine production by MDSC, which, in turn, influences M2 like-macrophage differentiation and stimulates the expansion of the immune-suppressive population. A recent paper shows for the first time that *in vivo* HLA-G skews the cytokine balance in favour of Th2 and stimulates expansion of MDSCs [125].

The interaction between HLA-G and monocytes due to ILT4 [126,127] inhibits maturation of human monocyte-derived APCs resulting in a reduced expression of MHC class II antigens and co-stimulatory molecules through Stat3 activation [128]. A study using human monocyte-derived DCs and LILRB inhibitory ILT4-transgenic mice shows that HLA-G induces the development of tolerogenic APCs with arrest maturation/activation of myeloid DCs and the gene expression profile provides evidence that HLA-G induces tolerogenic DCs by disruption of the MHC class II presentation pathway [129].

Moreover, HLA-G1 and HLA-G5 interacting *in trans* with ILT2 and ILT4 on the membrane surface of macrophages induces up-regulation of the same inhibitory receptors in circulating/infiltrating macrophages even in the absence of an on-going immune response. The HLA-G-dependent up-regulation of HLA-G receptors might increase the activation threshold of the macrophages thus limiting the capabilities of the immune system to initiate a response against HLA-G expressing tissues. Furthermore, it induces the sensitivity of macrophages to direct inhibition not only by tissue-expressed HLA-G, but also by classical HLA class I molecules [130].

All three major subtypes of classical MHC class I molecules (HLA-A, HLA-B, HLA-C) can shape the microenvironment interacting with LILR receptors and with KIR receptors.

The classic MHC class I (HLA A and HLA B) can interact, with varying degrees of strength, with ILT2 and ILT4 on the meanwhile recruited myeloid cells in relation to the HLA I polymorphisms and HLA I class conformation negatively conditioning the level of myeloid activation. The Beta2-microglobulin-free forms of HLA-C molecules can engage ILT4 turning down the macrophages activation level as well as LILRA1 and LILRA3 on the same cells pushing the M2 alternative polarization. In this way, TAMs could enrol new immune cells and amplify the tumour-associated population.

TAM surface expression of HLA-C molecules, even in the absence of peptide, could be sufficient to inhibit HLA-C specific NK cells [92,131] and non-MHC-restricted effector T cells [132] due to KIR2DL1/2/3.

Furthermore, HLA I class I molecules expressed on APCs negatively regulates NK-derived IFN-gamma secretion through the inhibitory receptor ILT2 [133]. Thus NK-derived IFN-gamma has a key role in polarization toward Th1 immune response [134] and it is critical for the induction of a tumour-specific cytotoxic T cell response [135].

Dietrich et al. show that ILT2 and TCR co-localize at the immunological synapse formed between T cells and antigen presenting cells expressing ligands for ILT2 and TCR on their surface. ILT2 on T cells could potentially function as an inhibitory co-receptor, first blocking binding of CD8 and second by bridging ILT2 into proximity with the TCR engaging the same peptide MHC-I [136]. However, there also exist other processes to inhibit T cell response: ILT2 could be efficiently transferred from monocytes to autologous T cells by trogocytosis and integrate within the plasma membrane of the acquirer T cells. Furthermore, the acquired receptors can access compatible signalling machinery within acquirer T cells and use it to signal and alter the functions of their new host cells [137].

The soluble HLA-G secreted or shed by TAMs mediates tolerance due to the induction of immune-suppressive T cells [138] through the engagement of the LILRB inhibitory receptors ILT2 and ILT4 on myeloid APCs [138] and the secretion of the anti-inflammatory cytokine TGF-beta1 by myelomonocytic cells [122]. TGFbeta1 is known to contribute to tolerance in peripheral lymphocytes [139].

HLA-G1 can be also acquired through membrane transfer or trogocytosis by CD4-positive T cells and CD8-positive T cells from APCs, like macrophages during the process of immunological synapse formation between TCR and MHC class II or I and or CD28 and B7 [140-142]. HLA-G trogocytosis depends on the activation status of the acquirer cells and, once activated, T cells efficiently capture APC-produced molecules regardless of their antigen specificity. T cells reverse temporarily their function from effectors to regulatory cells inhibiting T-proliferative responses or exhibit an enhanced suppression activity. This “emergency” suppression mechanism might be temporary, due to the limited lifespan of borrowed HLA-G1 at the cell surface. However, it might also be sufficient to delay immune reactions and give time for real regulatory cells to differentiate and take over [143]. Furthermore, HLA G1 transfected APC lines function as immune-inhibitory cells capable of turning off alloproliferative responses of PBMCs as well as purified CD4+ T cells and of inducing a long term antigen-specific unresponsiveness, thus promoting a long lasting immune ignorance/tolerance [144].

HLA-G1 can be shed as soluble HLA-G by HLA G1 transfected APC lines in the microenvironment inducing the apoptosis of activated CD8+ T cells and NK [144], through CD8 ligation [145] or by enhancing the Fas ligand expression on cytotoxic effectors and triggering Fas/Fas ligand mediated cell death [146]. It is noteworthy that HLA-G in contrast to other MHC class I antigens, can link KIR2DL4 on a minority of CD56bright peripheral NK cells [147]. The engagement of this receptor results in the activation of NK cells, not for cytotoxicity, but for cytokine and chemokine secretion with a pro-angiogenic purpose [96].

Moreover, TAMs could express non-classical HLA class I molecules such as HLA-E. CD94/NKG2A heterodimers constitute NK cell inhibitory receptors that recognize the molecule HLA-E. Their interaction results in the production of the immune-suppressive cytokines IL-10 and TGF-beta by NK cells [148] as well as in the protection of HLA-E expressing cells by NK and CTL-dependent lysis [101,102].

## Conclusion

**Figure 1:**
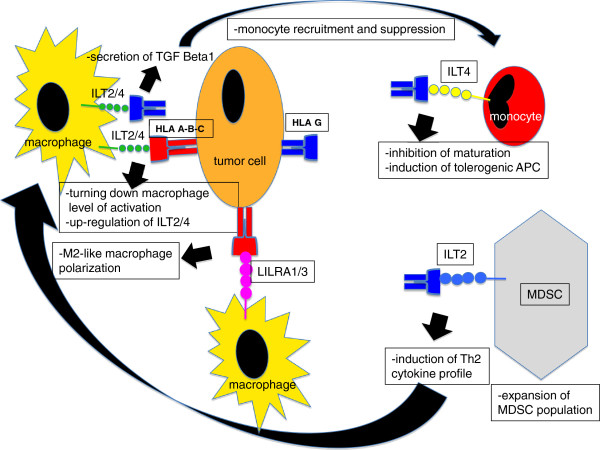
**Tumour cells express classical and non classical MHC class I.** The classic MHC class I, HLA A and HLA B, can interact, with varying degrees of strength, with ILT2 and ILT4 receptors on the myeloid cells in relation to the HLA polymorphisms and class conformation negatively conditioning the level of myeloid activation. The Beta2-microglobulin-free forms of HLA-C molecules are able to engage ILT4 turning down the macrophages activation level as well as LILRA1 and LILRA3 on the same cells pushing the M2 alternative polarization. The non classical MHC-I HLA G binds the inhibitory receptors ILT2 and ILT4 resulting in: macrophage production of TGF-beta1 resulting in a further recruitment of monocytes from blood circulation and suppression of their cytotoxic function; expansion of the population of myeloid derived suppressor cells (MDSC) with an enhanced suppressive activity; development of tolerogenic APC.

**Figure 2:**
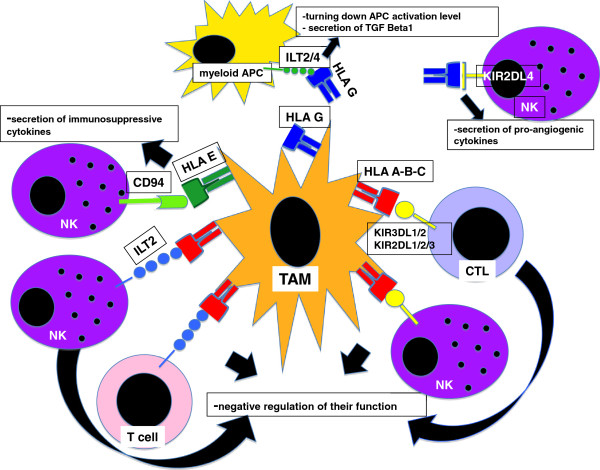
**TAMs express classical and non classical MHC class I.** The classic MHC class I HLA A, HLA B and HLA C can interact with ILT2 and ILT4 receptors on the myeloid cells with a different strength in relation to the HLA polymorphisms and conformation negatively conditioning the level of myeloid activation. HLA A, B and C inhibits NK and T cell function through the interaction with the inhibitory Killer cell immunoglobulin-like receptor KIR3DL1/2 and KIR2DL1/2/3 respectively. The non classical MHC-I, HLA-G, can link KIR2DL4 on a minority of NK cells resulting in the cytokine and chemokine secretion with a pro-angiogenic purpose. The non classical HLA E recognizes CD94/NKG2A on NK cells triggering the production of the immune-suppressive cytokines IL-10 and TGF-beta by NK cells as well as protecting TAMs by NK and CTL dependent lysis.

In conclusion, as summarized in Figures 1 and 2, both classical and non-classical MHC class I molecules expressed by tumour cells and TAMs might serve as important moderators of immune response attendees and the balance between the expression of MHC I molecules as free HC or as conformed proteins would determine the strength of such an immune response.

Indeed this review casts light on inhibitory and activatory LILRs as novel potential trigger factors of the tolerogenic profile and of M2-like alternative polarization on TAMs in response to the interaction with the classical and non classical MHC class I.

**Figure 3:**
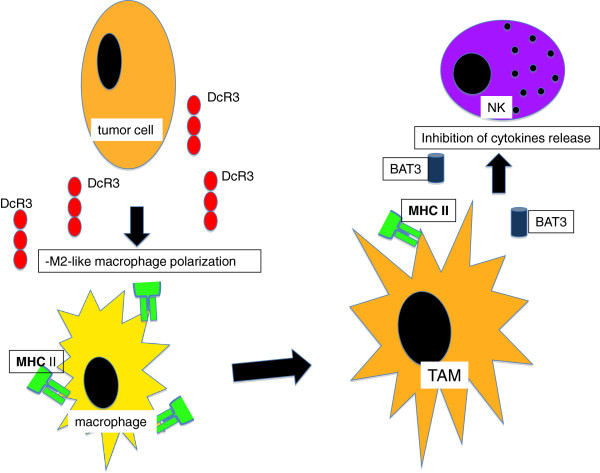
**Regulation of MHC class II expression on TAMs. **The tumour cells can secrete DcR3 that is shown to be a negative regulator of MHC class II expression and a promoter of M2-like macrophage polarization (TAM promotion). BAT3 is a critical intracellular factor for maintain MHC class expression on normal macrophages during the cell-mediated immune response. However TAMs are able to release it as a soluble immune-suppressive factor which inhibits NK cell-dependentcytokine secretion.

Other interesting potential targets of treatment represented in Figure 3, are DcR3 and BAT3.

The tumour cells can release DcR3 in the microenvironment, which not only decreases the MHC class II expression on macrophages, but also alternatively promotes their M2 polarization.

BAT3 is a critical factor for maintaining MHC class II expression on macrophage cell surfaces when acting on intracellular sites. However, when cancer and accessory cells, like TAMs, secrete BAT3 in the tumour microenvironment, this looks like acting as an immune suppressor factor.

## Abbreviations

APC: Antigen Presenting Cell; BAT3: HLA-B associated transcript-3; CD: Cluster of Differentiation; CMV: Cytomegalovirus; CTL: Cytotoxic T lymphocytes; CTLA: Cytotoxic T lymphocyte antigen; CIITA: Class II transactivator; DC: Dendritic cell; DcR3: Decoy Receptor 3; DNA: deoxyribonucleic acid; EGF: Epithelial Growth Factor; ER: Endoplasmatic Reticulum; FGF: Fibroblastic [Fibroblast?] Growth Factor; HIV: Human Immunodeficiency Virus; HLA: Human Leucocyte Antigen; ICOS: Inducible T-cell Costimulator; IL: Interleukin; ILT: Immunoglobulin-Like Transcript 2; INF: Interferon; iNOS: inducible NO Synthase; KIR: Killer cell Immunoglobulin-like Receptor; LILR: Leukocyte Immunoglobulin-like Receptor; LPS: Lipopolysaccarhide; M: Macrophage; MHC: Major Histocompatibility Complex; NSLC: Non-Small Lung Cancer; mRNA: messenger RiboNucleoAcid; MSC: Mesenchymal Stem cell; MDSC: Myeloid-derived Suppressor Cell; NHL: Non-Hodgkin Lymphoma; NK: Natural Killer; Stat: Signal transducer and activator of transcription protein; TAP: Transporter associated with antigen processing; TAM: Tumour-Associated Macrophage; TCR: T-Cell Receptor; TGF: Tumour Growth Factor; TNFR: Tumour Necrosis Factor Receptor; VEGF: Vascular Endothelial Growth Factor.

## Competing interests

The authors declare that they have no competing interests.

## Authors’ contributions

GVM is the project leader and he has designed the review, contributed with suggestions to the editing; MM has contributed to literature research and writing and concepts development; EA, LV, BS, AL and RK have contributed with specific editing and suggestion that regards their specific area of interest. All authors agreed and contributed to conceived the study, and participated to the final draft the manuscript. All authors read and approved the final manuscript.
